# Hemifusomes and proteolipid nanodroplets: a critical synthesis of an emerging model for endosomal membrane remodeling

**DOI:** 10.1186/s12964-026-03182-7

**Published:** 2026-09-09

**Authors:** Mohammed A. Abdel-Rasol, Wael M. El-Sayed

**Affiliations:** Department of Zoology, Faculty of Science, Ain Shams University, Abbassia, Cairo, Egypt

**Keywords:** Cryogenic electron tomography, Exosomes, Lipid-protein assemblies, Membrane biophysics, Membrane curvature, Multivesicular bodies

## Abstract

Vesicular membrane trafficking is central to eukaryotic homeostasis, governing receptor downregulation, lysosomal degradation, and intercellular communication via exosomes. These processes are classically explained by SNARE-mediated fusion and ESCRT-dependent membrane remodeling, which together define current models of vesicle biogenesis and multivesicular body (MVB) formation. Recent high-resolution cryo-electron tomography of intact mammalian cells has identified a structurally distinct membrane-associated assembly termed the hemifusome. Hemifusomes consist of two heterotypic vesicles connected by a persistent hemifusion diaphragm (~ 160 nm in diameter) and feature a ~ 42 nm proteolipid nanodroplet (PND) localized at the diaphragm rim. This architecture expands current views of hemifusion intermediates and raises the possibility of an alternative mode of endosomal membrane organization distinct from canonical ESCRT-mediated processes. However, the molecular composition, biogenesis, and regulatory mechanisms of this system remain undefined. Here, we provide the first integrated synthesis of hemifusome and PND biology, consolidating structural, biophysical, and mechanistic observations within the context of endosomal trafficking. We compare this emerging framework with ESCRT-mediated intraluminal vesicle formation, highlighting both shared features and key mechanistic gaps. We further examine preliminary and largely correlative links to neurodegenerative and lysosomal storage disorders, while emphasizing the absence of direct causal evidence. Given that this field currently rests on a single primary research report, we deliberately separate this evidence-based structural synthesis from more speculative functional, disease-related, and translational extensions, which are presented as explicit future perspectives rather than established conclusions. Finally, we outline unresolved questions, including PND molecular identity, determinants of hemifusion diaphragm organization and stability, and the temporal sequence of hemifusome assembly and remodeling. Together, these findings position hemifusomes as a potentially distinct structural state within the endosomal network, warranting systematic molecular and functional investigation.

## Introduction

Eukaryotic cellular homeostasis relies fundamentally on tightly regulated membrane dynamics that govern vesicle formation, fusion, and trafficking [1], which collectively underpin cellular communication and signaling control across intracellular compartments. Traditionally, these processes have been explained through two central mechanistic frameworks. The first is the SNARE-based model of membrane fusion, in which cognate SNARE proteins drive vesicle docking, hemifusion, and full membrane merger through progressive complex assembly [2]. The second is the ESCRT system, which mediates topologically reverse membrane remodeling events such as intraluminal vesicle (ILV) formation within endosomes through coordinated membrane deformation and scission reactions [3]. Together, these pathways form the conceptual foundation for multivesicular body (MVB) biogenesis, receptor downregulation, exosome formation, and lysosomal degradation, thereby directly shaping vesicle-mediated intercellular communication and signaling output.

Despite the robustness of these models, they do not fully account for all observed membrane intermediates in vivo. In particular, classical fusion theory predicts that hemifusion diaphragms (HDs) are transient, nanoscale structures (< 10 nm) that rapidly resolve into full fusion or scission events [4, 5]. Stable, large-scale hemifusion states in cellular systems were therefore considered biophysically unlikely, and intermediate stages of ILV formation remained largely inaccessible due to limitations of conventional fixation and imaging approaches [6, 7]. These constraints left open the possibility that additional, structurally distinct pathways of membrane remodeling may exist but remained undetected, potentially contributing to uncharacterized modes of endosomal signaling regulation.

This conceptual framework was fundamentally challenged in 2025 by Tavakoli et al., who employed high-resolution in situ cryo-electron tomography (cryo-ET) across multiple mammalian cell lines to identify a previously uncharacterized organelle complex termed the hemifusome [6]. Remarkably, hemifusomes constitute up to ~ 10% of peripheral endosomal structures in the examined systems and are defined by a stable hemifusion diaphragm averaging ~ 160 nm in diameter [4, 6]. This structure connects two heterotypic vesicles: a larger vesicle containing electron-dense, protein-rich luminal material and a smaller, protein-poor, translucent vesicle [6]. Two topological configurations were observed, including a “direct” cytoplasmic arrangement and a “flipped” form in which the smaller vesicle is internalized on the luminal side, resembling ILV-like topology without canonical ESCRT-mediated scission [6], suggesting a potential alternative route for regulating endosomal compartmentalization linked to vesicle-based communication processes.

A consistent and defining feature of hemifusomes is the presence of a ~ 42 nm electron-dense proteolipid nanodroplet (PND) localized at the rim of the hemifusion diaphragm [4, 6]. Situated within the hydrophobic core at the three-way membrane junction, the PND is hypothesized to stabilize membrane curvature and promote de novo vesicle formation, suggesting a potential ESCRT-independent mechanism of intraluminal vesiculation [6]. While these observations introduce a fundamentally new structural paradigm in membrane biology, their molecular basis, functional relevance, and physiological regulation remain unresolved, particularly in the context of how membrane architecture may influence endosomal signaling and communication states.

Importantly, despite the emergence of this novel system, a survey of the literature indicates that no dedicated reviews currently exist on hemifusomes or proteolipid nanodroplets. Only a limited number of original research articles are available (approximately four on hemifusomes and three on PND-related observations), highlighting the extreme nascency of the field. As such, there is currently no established conceptual synthesis integrating these findings into broader frameworks of membrane trafficking and biophysics, or into signaling-centered models of cellular communication. Given this narrow evidentiary base, we explicitly note that the present article adopts a narrative review structure to contextualize hemifusomes and PNDs within established membrane trafficking frameworks and to critically identify current knowledge gaps, while adopting an explicit Perspective orientation for discussion of functional, disease-related, and translational implications. Throughout this review, we distinguish direct structural and biophysical observations, derived from peer-reviewed cryo-electron tomography data, from mechanistic and translational extrapolations, which remain hypothetical and are consolidated in a dedicated Future Perspectives section rather than integrated into the evidence-based synthesis.

In this context, the present review provides the first review on hemifusome and PND biology. We contextualize these structures within established principles of membrane fusion, lipid organization, and ESCRT-mediated trafficking, and critically evaluate their proposed role as an ESCRT-independent pathway of vesicle formation. We further examine emerging, yet largely hypothetical, implications for multivesicular body maturation, exosome heterogeneity, and disease-associated endolysosomal dysfunction, with particular emphasis on their potential relevance to vesicle-mediated intercellular communication and signaling regulation. Finally, we identify the key mechanistic gaps that currently define the field, particularly the unknown molecular composition of PNDs, the lack of causal genetic evidence, and the absence of dynamic, temporal characterization of hemifusome formation and resolution.

## Methodology

This narrative review synthesizes the emerging evidence on hemifusomes and proteolipid nanodroplets (PNDs) as novel membrane-associated structures implicated in vesicular biogenesis and intracellular trafficking. Given the limited size of the current literature base, a focused and non-exhaustive search was conducted in PubMed for English-language publications published between 2010 and 2026. The search strategy was guided by Boolean combinations of relevant terms, including “hemifusome,” “proteolipid nanodroplet,” “hemifusion diaphragm,” “cryo-electron tomography,” “multivesicular body,” “vesicular biogenesis,” and “ESCRT-independent pathway.” Additional iterative searches were performed to ensure inclusion of mechanistically relevant studies on membrane remodeling, lipid-mediated vesiculation, and endosomal trafficking. Due to the nascent nature of the field and the small number of available studies, all directly relevant publications were included without the application of formal exclusion criteria. Instead, study selection was based on expert relevance assessment, prioritizing work that provided structural, mechanistic, or conceptual insight into hemifusome architecture, PND organization, or their proposed role in membrane dynamics. Data extraction focused on reported ultrastructural features, proposed mechanistic models, and functional interpretations to enable integrative synthesis across membrane biophysics and endosomal biology. Because primary experimental evidence is currently confined to a single research group and study, we have applied a stricter evidentiary standard when distinguishing “established observation” from “proposed interpretation” throughout the manuscript, and we reserve extended mechanistic or translational speculation for a clearly demarcated Future Perspectives section.

## Structural and biophysical characterization of hemifusomes and proteolipid nanodroplets

### Hemifusome ultrastructure

Hemifusomes were identified in thin peripheral regions (~ 250–450 nm ice thickness) of mammalian cells using in situ cryo-electron tomography (cryo-ET) [4, 6]. They consist of two heterotypic vesicles connected by a continuous hemifusion diaphragm (HD) [6]. The larger vesicle (mean radius ~ 299 ± 96 nm) displays electron-dense luminal content typical of endosomes, whereas the smaller vesicle is translucent and protein-poor [4, 6].

Topologically, hemifusomes exhibit merged exoplasmic leaflets forming the HD, while cytoplasmic leaflets remain continuous with the cytosol (~ 4 nm thickness) [6, 8]. This configuration is consistent with classical hemifusion intermediates, yet differs fundamentally in scale and persistence, suggesting that hemifusomes are not transient intermediates but stabilized structures with potential implications for membrane-based communication and trafficking regulation within the endosomal system.

A defining feature is the HD diameter (~ 160 ± 61 nm), far exceeding the sub-10 nm diaphragms predicted by canonical fusion models [4, 6]. This discrepancy indicates that current fusion paradigms are insufficient to explain hemifusome stability. We interpret this as evidence for an additional stabilizing mechanism rather than an extension of classical hemifusion within pathways that may intersect with endosomal signaling and vesicle-mediated communication. HD curvature variability (bulged, flat, inverted) implies regulation by membrane tension, lipid composition, and cytoskeletal forces [4, 6], although causality remains untested. The “flipped” configuration, where the smaller vesicle is internalized, resembles intraluminal vesicles (ILVs) but differs in its persistent HD connection [6], suggesting a potential intermediate state in vesicle-based communication processes rather than canonical ESCRT-driven maturation.

Approximately 25% of hemifusomes form compound assemblies [6], indicating structural robustness. However, the absence of functional correlates (e.g., trafficking, signaling) limits interpretation of their biological role. Hemifusomes represent a structurally well-defined but functionally unresolved entity, with current evidence supporting their existence but not yet their physiological significance in cellular communication networks governed by endosomal signaling pathways.

### Structure and role of proteolipid nanodroplets

Proteolipid nanodroplets (PNDs) are ~ 42.4 ± 4.3 nm electron-dense structures consistently localized at the HD rim. Positioned within the bilayer core, they interface with both vesicles and the HD [4, 6]. Their phase-dark interior suggests a hydrophobic lipid core, while discrete densities indicate protein content [6, 9, 10]. This supports a hybrid proteolipid identity distinct from classical lipid droplets. However, this conclusion is inferred solely from morphology, as no compositional analyses have been performed.

PND-like structures are also observed as free cytoplasmic particles and intramembrane inclusions [4, 6], forming the basis of a proposed nucleation model. While their spatial distribution is consistent with this possibility, no direct temporal or mechanistic evidence currently demonstrates a nucleation function. PNDs are consistently localized at the rim of the hemifusion diaphragm, a highly curved membrane junction where geometric strain is expected to be maximal. This spatial association supports the possibility that PNDs may influence membrane curvature; however, it remains equally possible that they are passively recruited to pre-existing curved membrane regions rather than actively generating or stabilizing curvature. Therefore, the current evidence supports a curvature-regulatory hypothesis but does not establish a causal role.

PND localization is not random with respect to hemifusome architecture: across reported observations, PNDs are consistently positioned at the rim of the HD, the specific site where the merged exoplasmic leaflets transition into the two topologically distinct cytoplasmic leaflets of the flanking vesicles [4, 6]. This rim constitutes a three-way membrane junction and, by geometric necessity, represents a region of high local curvature and membrane strain within the hemifusome, as classical bilayer packing may be challenged by the sharply bent, toroidal leaflet geometry at this interface [6, 11, 12]. The preferential association of PNDs with this region has led to the hypothesis that they may contribute to local membrane curvature regulation or stabilization. Mechanistically, such a role could involve accommodation of curvature-associated packing constraints, analogous to curvature-modulating principles observed for amphipathic wedge insertion by BAR-domain proteins or enrichment of conical lipids. However, whether PNDs actively generate or stabilize curvature, or instead preferentially accumulate at already curved membrane regions, remains unresolved.

The near-invariant ~ 42 nm PND diameter and consistent localization at the HD rim provide structural observations that motivate, but do not prove, a functional role in curvature regulation. The reproducibility of these features across reported hemifusome observations suggests that PND positioning may be structurally constrained rather than incidental. Nevertheless, direct measurements of membrane energetics, curvature changes following PND perturbation, or reconstitution studies are required to determine whether PNDs actively contribute to membrane bending or simply associate with pre-existing high-curvature domains. Thus, PNDs should currently be considered structurally defined but mechanistically unresolved components of hemifusomes, with their proposed role in membrane curvature remaining a testable hypothesis. The absence of molecular identity represents the single largest barrier to mechanistic understanding and reproducibility and currently limits their integration into established signaling and trafficking frameworks within cellular communication systems.

### Comparison with related organelles

Hemifusomes display a topology not observed in canonical organelles. Unlike endosomes and lysosomes, which are closed bilayer compartments [13], hemifusomes maintain a shared exoplasmic leaflet and cytosol-facing inner leaflets [6]. The smaller vesicle resembles ILVs in size and composition [13], but differs critically in topology and fate. ILVs undergo ESCRT-mediated scission, whereas hemifusome vesicles are observed by cryo-ET as hemifused vesicle pairs that remain connected via an HD over the imaging timescale [4, 6]. In the current model, this HD-mediated connection is interpreted as a metastable intermediate capable of topological remodeling, including transitions between direct and flipped configurations and eventual remodeling toward sealed intraluminal vesicles, rather than as a terminal stable state [4, 6]. These trajectories are inferred from an ensemble of static structural snapshots rather than direct dynamic observation and should therefore be regarded as a proposed mechanistic framework for hemifusome behavior rather than an experimentally established sequence of events.

The proposed ESCRT independence is based on absence of transferrin receptor labeling [6, 13]. While suggestive, this is insufficient evidence, as it relies on a single cargo marker and does not exclude partial or context-dependent ESCRT involvement or broader signaling-dependent regulation of vesicle sorting. Similarly, PNDs differ from classical lipid droplets in size and bilayer embedding [6, 14], but these distinctions remain morphological.

Compared with the ESCRT system, which is mechanistically and molecularly well established, hemifusomes are currently defined primarily at the structural level. This imbalance highlights their early stage of mechanistic maturity and underscores the need for molecular and functional validation to determine their role, if any, in endosomal signaling and intercellular communication pathways.

### Biophysical basis of HD formation and stability

Membrane hemifusion progresses through stalk formation and expansion into an HD [6]. Membrane tension and lipid composition govern these transitions [15, 16]. However, classical models predict transient HDs, whereas hemifusomes exhibit large, persistent diaphragms. This mismatch indicates that additional stabilizing factors are required.

Based on the primary cryo-ET study, PNDs may act as localized structural elements at the HD rim that could contribute to reducing the energetic constraints associated with maintaining highly curved membrane interfaces [6, 11]. In this context, “line tension buffering” is best understood as a biophysical interpretation of the observed rim-localized PND geometry rather than a directly measured mechanism. This model provides a plausible explanation for how PNDs may influence HD organization; however, it remains experimentally unvalidated and should be considered a structural hypothesis rather than an established mechanism. Any potential consequences for membrane-associated signaling environments remain speculative.

Comparisons to viral fusion proteins and SNARE systems highlight similar rim-localized stabilization [5, 17]. However, these systems are transient and protein-driven, whereas hemifusomes appear stable and structurally persistent. Hemifusomes likely represent a stabilized hemifusion state, distinct from classical fusion intermediates with potential relevance to regulated vesicle-based communication in endosomal pathways.

## Biogenesis of hemifusomes

### Models of hemifusome formation

**Fig. 1:**
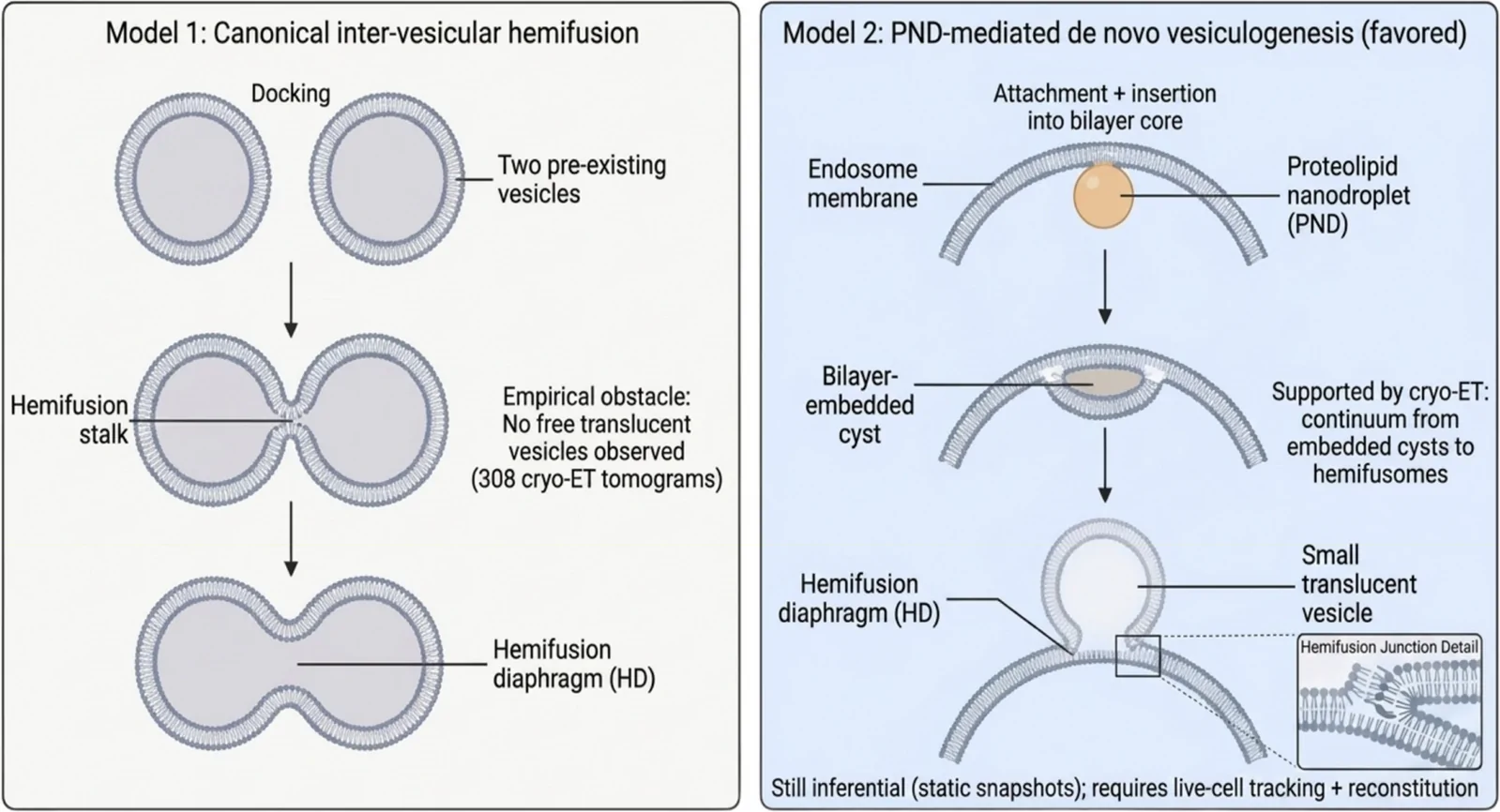
Models of hemifusome formation: canonical inter-vesicular hemifusion versus De Novo Vesiculogenesis. Abbreviations: cryo-ET: Cryo-electron tomography, HD: Hemifusion diaphragm, PND: Proteolipid nanodroplet

Two models are proposed: (i) vesicle-vesicle hemifusion and (ii) PND-mediated de novo vesiculogenesis. The classical model aligns with established fusion theory [18] but is weakened by the absence of observable precursor vesicles in cryo-ET datasets [6]. While this argues against the model, it does not exclude transient intermediates below detection thresholds. The de novo model posits that PNDs insert into endosomal membranes to nucleate vesicle formation [6, 19]. Morphological continuums support this [6, 20], but these observations are static and inferential. Neither model is definitively validated; current evidence favors the de novo model but remains insufficient (Fig. 1) for defining a mechanistically resolved pathway within endosomal communication systems.

### Relationship to ESCRT pathways

Both ESCRT-mediated ILV formation and hemifusomes generate intraluminal vesicle-like structures [6], suggesting potential functional overlap. However, they differ in topology and proposed mechanism. ESCRT pathways involve cytoplasmic membrane deformation and scission [21], whereas hemifusomes contain a persistent HD that separates the paired vesicles without full luminal continuity or fusion pore opening [6]. The absence of transferrin uptake is suggestive of divergence from classical transferrin-positive endosomal and recycling compartments [6, 22], but it does not by itself establish ESCRT independence. This interpretation remains provisional, because negative cargo-marker data cannot exclude partial, context-dependent, or stage-specific ESCRT contributions, nor can it exclude involvement of auxiliary adaptors or pathway cross-talk. A more rigorous assessment of ESCRT involvement will require direct perturbation of core ESCRT components, such as ESCRT-0/ESCRT-I/ESCRT-III factors or Vps4, combined with quantitative analysis of hemifusome frequency, HD morphology, and PND localization, together with rescue experiments and cargo-tracking approaches.

**Fig. 2:**
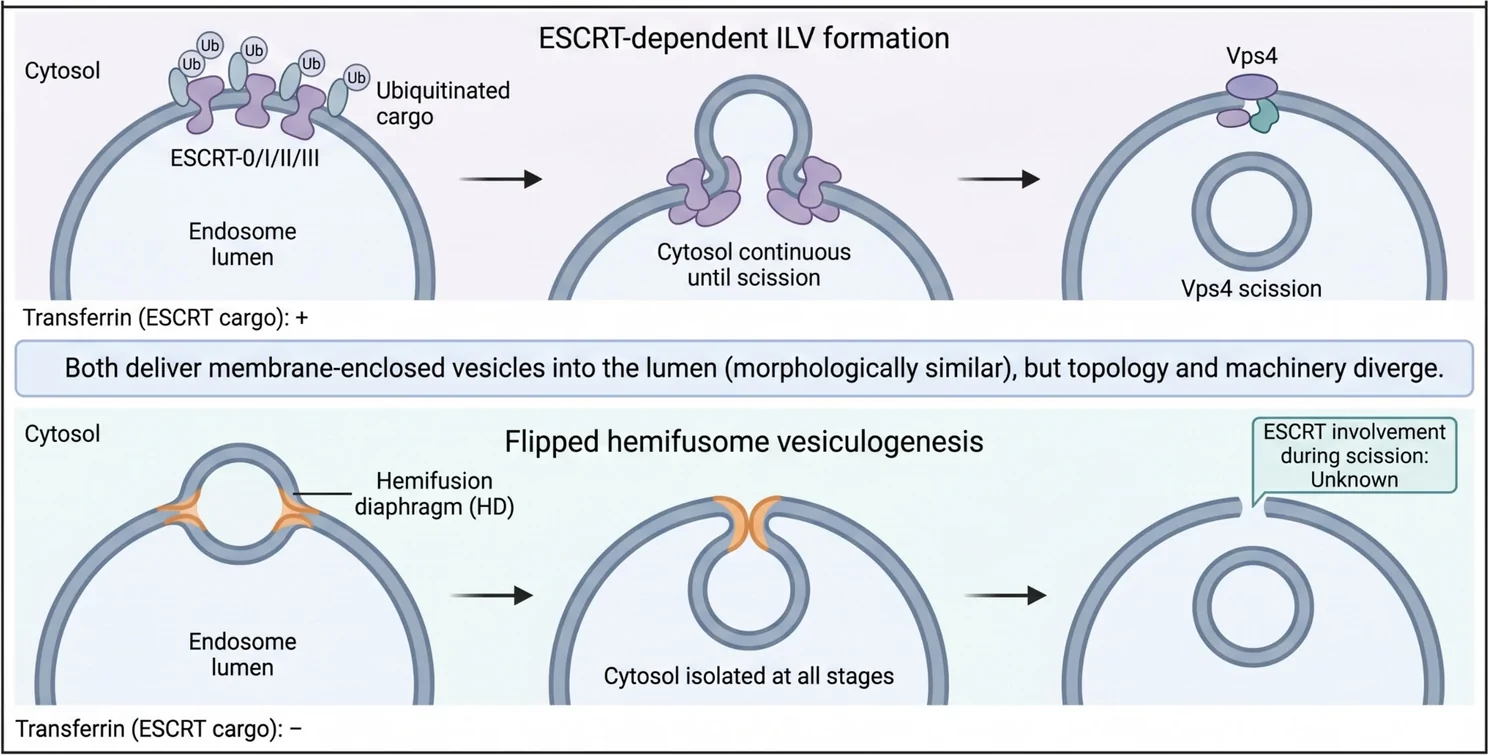
Topological and mechanistic distinctions between ESCRT-Mediated ILV formation and hemifusome biogenesis. Abbreviations: ESCRT: Endosomal sorting complexes required for transport, HD: Hemifusion diaphragm, Vps4: Vacuolar protein sorting 4

Mechanistically, hemifusomes could therefore represent an alternative trafficking pathway, a specialized adaptation of existing membrane remodeling processes, or a context-dependent divergence from ESCRT-mediated mechanisms. However, current evidence is insufficient to distinguish among these possibilities, leaving their precise classification unresolved (Fig. 2) within the broader framework of cellular communication and endosomal signaling networks.

### Molecular players: confirmed and candidate

PNDs are ~ 42 nm proteolipid assemblies with defined spatial localization [6], but their molecular composition remains unknown. Cytoskeletal interactions suggest a possible mechanical influence of actin and microtubules on hemifusome morphology, although the available evidence mainly supports physical proximity or potential local deformation rather than direct regulation [6, 23]. Proposed lipid and protein contributors are biologically plausible but have not been experimentally validated [6, 21, 24]. The reported one-to-one PND-hemifusome association suggests a possible structural relationship; however, whether this reflects a nucleation function or another form of spatial organization remains unresolved. Functional links between PNDs and signaling or trafficking regulation remain speculative.

### PNDs in curvature generation and HD resolution

The HD rim represents a highly curved membrane region requiring stabilization of local membrane organization [4, 12]. PND localization at this interface supports the hypothesis that they may contribute to curvature regulation or buffering of membrane stress [4, 6]. One proposed interpretation is that PNDs may act as mechanical stabilizing elements that influence the persistence of the hemifused state without necessarily driving progression to full fusion. In this context, “HD resolution” refers to the morphological remodeling or apparent disappearance of the hemifusion diaphragm in the flipped hemifusome configuration, rather than to a directly observed scission event. In flipped hemifusomes, this remodeling is associated with a luminal topology consistent with an ILV-like state, but the sequence and mechanism of any transition remain unresolved in the available cryo-ET data [4, 6]. Accordingly, ILV release should be regarded as a structural inference rather than an established functional outcome.

### Synthesis and conceptual positioning

Hemifusomes are structurally well-supported but mechanistically immature entities. Their defining features, large stable HDs and PND localization, challenge existing membrane fusion paradigms. While current models are plausible, they remain largely inferential. Advancing the field requires transitioning from structural description to molecular and functional validation within a framework that integrates membrane architecture with cellular communication and endosomal signaling dynamics.

## Functional implications of hemifusomes in cellular physiology and disease

### Cargo sorting, lipid distribution, and MVB maturation

Hemifusomes constitute approximately 10% of the endolysosomal organelle population across mammalian cell types [6, 25], suggesting that they are not rare structural anomalies but potentially represent a regulated membrane remodeling state within endosomal trafficking networks that interface with cellular communication pathways. However, this prevalence alone does not establish function; rather, it raises a key interpretational challenge: whether hemifusomes are active participants in endosomal trafficking or structurally stabilized intermediates with limited physiological turnover.

Mechanistically, hemifusome-associated de novo vesiculogenesis is proposed to operate as a parallel pathway to ESCRT-dependent intraluminal vesicle (ILV) formation [6]. In this model, maturation of the smaller vesicle followed by hemifusion diaphragm (HD) resolution would yield sealed intraluminal compartments, thereby contributing to multivesicular body (MVB) expansion [6, 26], with potential downstream consequences for endosomal signaling output and intercellular communication via vesicle release. While this framework is consistent with observed morphologies, it remains inferential due to the absence of dynamic tracking of vesicle maturation.

Cargo specificity within this pathway is currently unresolved. The exclusion of transferrin receptor labeling suggests divergence from canonical ESCRT cargo handling [6, 26], but this evidence is limited and does not exclude broader overlap with endocytic trafficking networks. The protein-poor nature of the nascent vesicle further suggests possible enrichment in lipids, whereas any inference regarding non-ubiquitylated components remains speculative and is not directly demonstrated in the primary study [6, 26, 27]. As such, compositional interpretation should presently be limited to the structural observation of relative protein paucity, pending direct molecular profiling.

The proteolipid nanodroplet (PND), positioned at the HD rim, is hypothesized to contribute to lipid redistribution and membrane remodeling by acting as a localized lipid reservoir [6], potentially influencing membrane composition states that are known to regulate signaling-associated endosomal sorting processes. This is conceptually attractive given its invariant localization, yet no direct measurements of lipid flux or enzymatic activity have been performed. As such, its functional role should currently be considered a working hypothesis rather than an established mechanism.

Overall, hemifusome-mediated contributions to cargo sorting and MVB maturation are structurally supported but mechanistically unvalidated, placing this system at an early stage of functional characterization (Table 1), particularly in relation to its potential role in endosomal signaling regulation and vesicle-mediated communication dynamics.

**Table 1:** Proposed functional implications and mechanisms of hemifusomes in cellular physiology

Cellular Process / Feature	Proposed Mechanism and Observations	Current Limitations and Evidence Gaps	References
Organelle Prevalence	Comprise ~ 10% of mammalian endolysosomal organelles, indicating a regulated membrane remodeling state rather than a rare structural anomaly.	Unclear whether they act as active participants in endosomal trafficking or structurally stabilized intermediates with limited physiological turnover.	[6, 25]
Vesiculogenesis and MVB Maturation	Operates as a parallel pathway to ESCRT-dependent ILV formation via *de novo* vesiculogenesis. Maturation of smaller vesicles and HD resolution yield sealed intraluminal compartments, driving MVB expansion.	Mechanism remains inferential; lacks dynamic tracking of vesicle maturation to confirm structural progression.	[6, 26]
Cargo Sorting Specificity	Diverges from canonical ESCRT handling, evidenced by the exclusion of transferrin receptor labeling. Protein-poor nascent vesicles suggest enrichment in lipids or non-ubiquitylated components.	Highly speculative; requires direct proteomic or lipidomic profiling to resolve true specificity and network overlap.	[6, 26]
Lipid Distribution	PNDs consistently localized at the HD rim act as hypothesized localized lipid reservoirs to facilitate lipid redistribution and membrane remodeling.	Constitutes a working hypothesis; no direct measurements of lipid flux or enzymatic activity have been conducted.	[6]

*Abbreviations*: *ESCRT* Endosomal Sorting Complexes Required for Transport, *HD* Hemifusion Diaphragm, *ILV* Intraluminal Vesicle, *MVB* Multivesicular Body, *PND* Proteolipid Nanodroplet

### Intersection with exosome biogenesis

Multivesicular bodies serve as a critical sorting hub, where internal vesicles are either degraded via lysosomes or secreted as exosomes [27]. In this context, hemifusome-derived ILVs could theoretically contribute to the heterogeneity of extracellular vesicle populations [6, 28], thereby linking membrane remodeling states to variability in intercellular communication outputs. However, this connection remains hypothetical, as direct evidence linking hemifusomes to exosome secretion is currently absent.

Despite this limitation, morphological similarity between hemifusome-derived vesicles and exosome precursors suggests a plausible mechanistic intersection [6, 29]. If hemifusome-derived ILVs enter the secretory MVB pathway, their distinct biogenetic origin could influence exosomal cargo composition, thereby contributing to functional heterogeneity in intercellular communication systems [30].

A key unresolved question is whether PND insertion imparts a stable proteolipid signature that persists after vesicle maturation. While this is a reasonable hypothesis based on structural continuity, it has not been experimentally demonstrated. Therefore, the proposed contribution of hemifusomes to exosome diversity should be viewed as a mechanistic possibility rather than an established pathway, pending validation within experimentally defined communication networks of extracellular vesicles.

Resolving this will require integration of correlative cryo-electron tomography with single-vesicle lipidomic and proteomic analyses capable of distinguishing ILV subpopulations by origin.

### Speculative links to disease-associated endolysosomal dysfunction

Dysfunction of the endolysosomal system is a well-established early event in multiple neurodegenerative disorders [31], raising the possibility that hemifusomes may intersect with disease-associated membrane trafficking disturbances that influence cellular communication and signaling homeostasis. However, because direct evidence linking hemifusomes, PNDs, or HD stability to disease phenotypes is currently lacking, these relationships should be regarded as speculative connections to broader endolysosomal dysfunction rather than established disease mechanisms.

In Alzheimer’s disease, early endosomal enlargement is linked to impaired ILV formation, defective retromer recycling, and altered endosomal acidification [32]. If future studies demonstrate a significant contribution of hemifusomes to ILV biogenesis, disruption of PND-associated vesiculogenesis or HD organization could represent a potential mechanism influencing these pathological changes. However, no direct evidence currently connects hemifusomes to Alzheimer’s disease progression.

In Parkinson’s disease, mutations in PRKN have been associated with altered vesicle trafficking and increased exosome release [33]. While it is possible that some trafficking alterations could intersect with hemifusome-associated processes, this remains untested and should be interpreted cautiously.

Similarly, lysosomal storage disorders exhibit profound MVB abnormalities due to lipid accumulation and defective hydrolase activity [34]. Given the proposed involvement of lipid-associated PND structures, altered lipid homeostasis could potentially influence hemifusome organization or dynamics; however, this connection remains hypothetical and requires experimental validation. Future studies should therefore quantify hemifusome abundance by cryo-ET in disease-relevant models, assess whether altered lipid homeostasis changes PND composition, and test whether perturbation of trafficking regulators affects HD stability or topological remodeling (Table 2).

**Table 2:** Proposed disease relevance and pathological implications of hemifusomes

Disease Context	Established Pathological Phenotypes	Hypothesized Hemifusome Involvement	Current Evidence Level and Limitations	References
General Neurodegenerative Disorders	Endolysosomal system dysfunction acting as an early pathological event.	May contribute generally to disease-associated membrane trafficking defects.	Speculative but mechanistically plausible; lacks data linking hemifusome abundance, PND composition, or HD stability to pathological phenotypes.	[31]
Alzheimer’s Disease	Early endosomal enlargement linked to impaired ILV formation, defective retromer recycling, and altered endosomal acidification.	Disruption of PND-mediated vesiculogenesis or HD stability could exacerbate early pathological changes (if hemifusomes significantly contribute to ILV biogenesis).	No direct evidence currently connects hemifusomes to disease progression.	[32]
Parkinson’s Disease	*PRKN* mutations associated with altered vesicle trafficking and increased exosome release.	Disease phenotypes may reflect the underlying dysregulation of hemifusome activity.	Completely untested; remains a speculation requiring cautious interpretation.	[33]
Lysosomal Storage Disorders	Profound MVB abnormalities caused by lipid accumulation and defective hydrolase activity.	Altered lipid homeostasis may indirectly affect hemifusome dynamics, particularly concerning the proposed lipid-rich PND structures.	Currently hypothetical; strict biochemical validation is required.	[34]

*Abbreviations*: *HD* Hemifusion Diaphragm, *ILV* Intraluminal Vesicle, *MVB* Multivesicular Body, *PND* Proteolipid Nanodroplet

### Emerging translational questions

Beyond their structural characterization, hemifusomes and PNDs raise potential translational questions related to intracellular delivery and biomarker discovery; however, these concepts currently lack direct experimental support and are introduced as hypothesis-generating possibilities rather than established applications.

Despite limited mechanistic understanding, hemifusomes and PNDs may provide a conceptual framework for future bioengineered membrane-remodeling systems within the broader context of modulating intracellular communication pathways. The membrane-associated and curvature-related properties of PNDs are structurally intriguing, but current evidence does not demonstrate that PNDs can nucleate vesicle formation around exogenous cargo, bypass conventional endocytic barriers, or mediate intracellular delivery. Comparisons with lipid nanoparticle platforms and their endosomal escape limitations [35, 36] should therefore be interpreted as conceptual parallels rather than evidence that hemifusome-inspired systems currently represent a practical alternative. At present, no synthetic PND-like particle has been engineered, and critical parameters including cargo loading, membrane targeting, delivery efficiency, and safety remain untested. Establishing even a proof-of-concept platform would require independent validation of each of these properties before any translational potential could be assessed.

From a diagnostic perspective, if PND-specific molecular components are identified, they may represent candidate biomarkers reflecting alterations in endosomal membrane dynamics [37] and potentially provide indicators of altered cellular communication states in disease. This concept is analogous to exosomal tetraspanins but would represent a distinct axis of endosomal biology. The major limitation is that no molecular signature of PNDs has yet been defined. Advancing either therapeutic or diagnostic applications will require molecular identification of PND components and development of approaches to experimentally manipulate hemifusome formation in vivo, which currently represents a major technological challenge.

## Hemifusomes at the interface of nanotechnology and synthetic biology

### Bio-inspired nanoparticle design

**Fig. 3:**
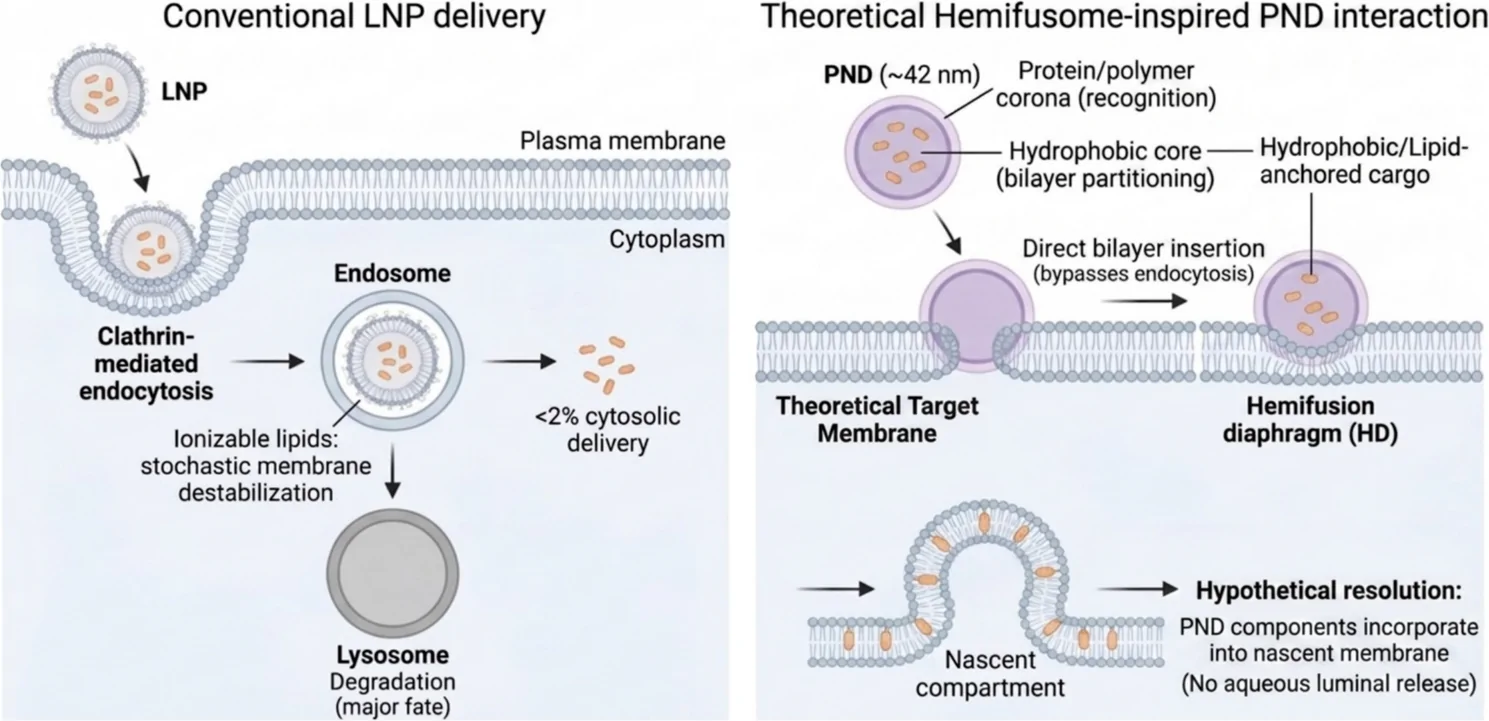
Bio-inspired nanoparticle design versus conventional lipid nanoparticle (LNP) delivery. Abberivations: HD: Hemifusion diaphragm, LNP: Lipid nanoparticle, PND: Proteolipid nanodroplet

Hemifusomes and PNDs provide a structural framework for exploring future concepts in intracellular delivery system design [6], by highlighting potential relationships between membrane architecture, intracellular trafficking, and communication behavior. Current lipid nanoparticle platforms rely primarily on stochastic endocytosis and inefficient endosomal escape, resulting in limited cytosolic delivery efficiency [35, 36]. In contrast, hemifusome biology raises a conceptual possibility that membrane-associated remodeling within intracellular vesicular compartments could influence compartmental organization or cargo fate after uptake. However, the available evidence does not show that PNDs traverse the plasma membrane, insert into the plasma membrane, bypass endocytic uptake, or mediate cargo transfer. Accordingly, any delivery-related interpretation should be restricted to hypothetical post-endocytic processes, such as altered endosomal confinement or vesicular remodeling after internalization, rather than direct cell entry or demonstrated cytosolic delivery. This remains a conceptual extrapolation from structural observations. No synthetic system has yet reproduced PND-like behavior in living cells, and therefore its applicability to nanomedicine should be considered a hypothesis rather than a validated technology (Fig. 3).

### Engineering artificial hemifusomes and PNDs

Artificial induction of membrane hemifusion in vitro is well established using giant unilamellar vesicles and curvature-inducing lipid compositions [38, 39]. However, stabilizing hemifusion diaphragms at biologically relevant scales (~ 100–200 nm) remains a major unresolved engineering challenge. A central hypothesis derived from hemifusome biology is that stabilization requires a nanoscale hydrophobic particle positioned at the rim of the hemifusion diaphragm to regulate line tension [6]. Synthetic analogs such as amphiphilic nanoparticles, Janus particles, or polymer-lipid hybrids may approximate this function, but their ability to reproduce PND-like insertion behavior remains unproven.

Although fabrication of lipid nanodroplets in the 30–80 nm range is technically feasible, achieving true PND functionality requires more than size control; it requires controlled membrane insertion and curvature sensing, which currently lacks experimental validation. As such, artificial PND construction remains at a proof-of-concept stage.

### Conceptual basis for hemifusome-inspired drug delivery

We introduce this concept here as a structural rationale derived from Sect.  "Bio-inspired nanoparticle design", "Engineering artificial hemifusomes and PNDs"; a fuller, explicitly speculative discussion of its translational implications is reserved for the Future Perspectives section, consistent with the current absence of experimental drug-delivery data derived from hemifusome or PND biology. A major limitation in intracellular drug delivery is entrapment within endosomal compartments [40]. Hemifusome-inspired systems represent a hypothetical concept in which post-uptake membrane remodeling could potentially influence cargo fate within intracellular vesicular compartments associated with cellular communication pathways.

In theory, topological remodeling of a hemifusion intermediate could alter the organization or accessibility of cargo within intracellular compartments [39, 41]. However, this should be regarded as a speculative possibility rather than a demonstrated mechanism for cytosolic cargo release. Current evidence does not show that hemifusomes directly mediate endosomal escape, and the precise mechanisms by which cargo could move from a hemifusion-associated compartment to the cytosol remain undefined.

**Fig. 4:**
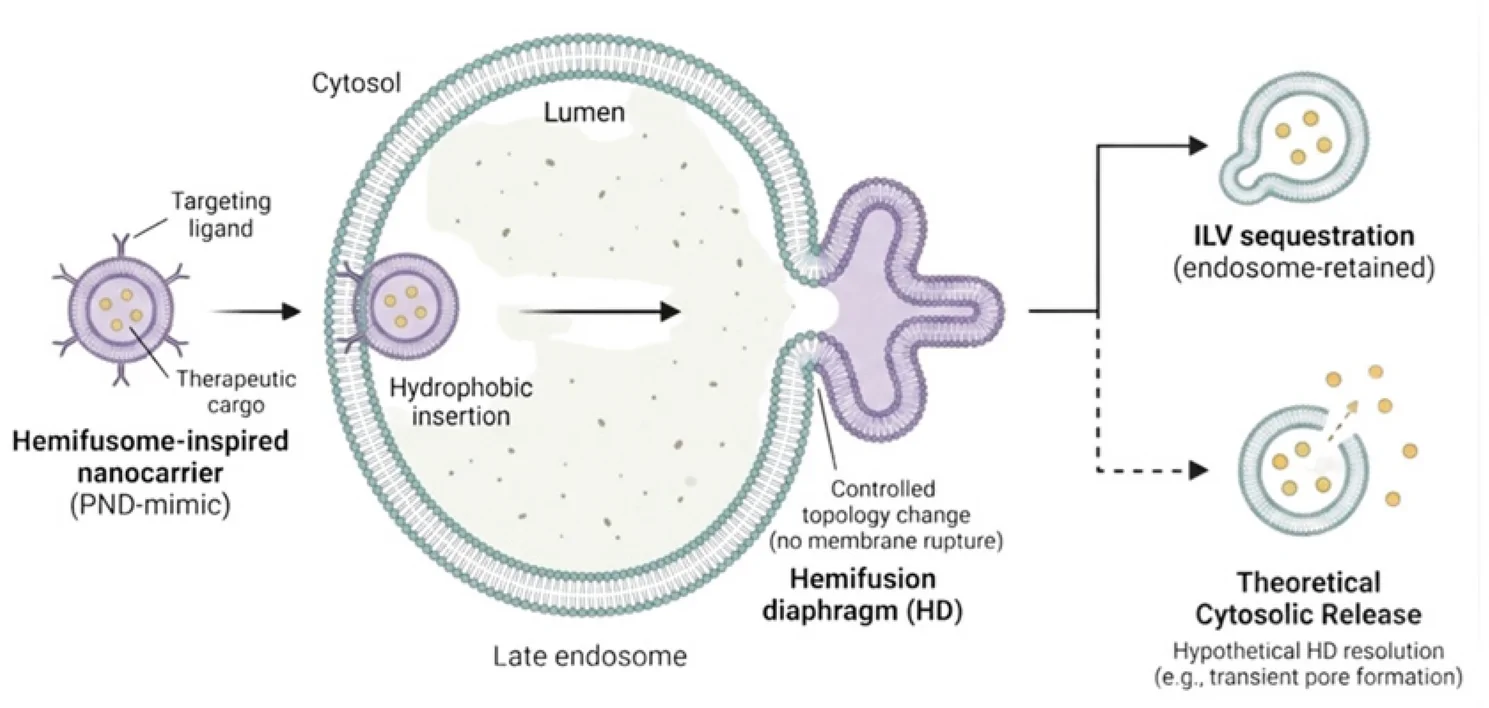
Hemifusome-inspired nanocarriers: mechanisms for biphasic endosomal escape and organelle-targeted drug delivery. Abbreviations: HD: Hemifusion diaphragm, PND: Proteolipid nanodroplet

An additional unresolved factor is the apparent selectivity of PND localization to specific endosomal states, suggesting a possible relationship with local membrane composition or curvature-associated molecular interactions [6, 42]. While this represents a compelling hypothesis, no molecular determinants have been identified. Future design strategies may explore targeting modules such as Rab-binding peptides or phosphoinositide-recognition domains; however, these remain speculative possibilities rather than validated design principles (Fig. 4).

### Artificial organelles as synthetic biology modules

Hemifusomes introduce a broader conceptual advance: the possibility that stable hemifusion interfaces can serve as templates for intracellular compartment engineering [6, 43], expanding synthetic biology frameworks toward systems that interface directly with intracellular communication networks. This positions them as a potential framework for constructing programmable membrane-based organelles. While synthetic biology has successfully generated artificial organelles such as engineered peroxisomes and lipid compartments [43, 44], hemifusomes suggest an additional design principle based on stabilized membrane intermediates rather than fully sealed compartments.

A hypothetical artificial hemifusome module would integrate lipid-based cores, curvature-stabilizing elements, and stimuli-responsive scission mechanisms. However, such a system has not yet been constructed, and its feasibility remains theoretical.

The broader implication is that hemifusomes may serve less as directly translatable structures and more as conceptual models for designing membrane systems with tunable stability and topological plasticity, and as conceptual intermediates for understanding how membrane architecture can regulate cellular communication states. Realizing this vision will require substantial advances in synthetic membrane engineering and real-time structural validation.

Across physiological, pathological, and engineering contexts, hemifusomes are best interpreted not as fully understood functional organelles but as a stabilized hemifusion state with emergent regulatory potential. Their significance lies less in confirmed biological roles and more in the mechanistic challenge they pose to existing models of membrane remodeling. This positions them as a transitional conceptual bridge between classical vesicle biology and programmable membrane systems that intersect with endosomal signaling and intercellular communication biology.

## Current challenges and knowledge gaps in hemifusome research

### Limite cell-type coverage

Although hemifusomes have been consistently observed in four mammalian cell lines (COS-7, HeLa, RAT-1, NIH/3T3) [6, 25], the current dataset is heavily biased toward proliferative, adherent cell types. This introduces a fundamental limitation: the apparent 10% organellar prevalence may reflect a context-dependent phenomenon rather than a universal cellular feature across diverse signaling and communication states of mammalian tissues. Critically, major physiological systems, including neurons, immune cells, secretory epithelia, and highly polarized tissues, remain entirely unexplored. This omission constrains interpretation of hemifusomes as either a general membrane remodeling mechanism or a lineage-restricted adaptation with potential roles in cell-type-specific communication pathways.

At present, the field lacks evidence to determine whether hemifusomes are evolutionarily conserved across differentiated cell states or dynamically regulated according to cellular function and signaling demands. Resolving this requires systematic ultrastructural profiling across primary tissues and developmental stages.

### The perturbation gap

The most significant limitation in current hemifusome research is the absence of causal perturbation studies. All mechanistic interpretations are derived from static cryo-electron tomography (cryo-ET), meaning that the field remains entirely structure-driven rather than functionally validated. No genetic or pharmacological manipulation has been shown to alter hemifusome formation, HD morphology, or PND localization. Consequently, all proposed biogenetic models remain inferential, regardless of internal morphological consistency.

Although the absence of transferrin receptor labeling supports ESCRT independence [6], this represents negative evidence and does not establish alternative machinery. Without gene-level disruption, it remains impossible to determine whether hemifusomes are required for MVB maturation, ILV production, or exosome release and therefore for modulation of endosomal signaling outputs and vesicle-mediated communication. This represents a fundamental maturity gap: hemifusomes are structurally defined but not yet experimentally causal entities. Bridging this gap is the central prerequisite for advancing the field from descriptive morphology to mechanistic cell biology within a signaling-relevant framework of endosomal function.

### Unknown PND biochemical composition

Despite being central to all proposed mechanisms, the proteolipid nanodroplet (PND) remains biochemically undefined. Neither its lipid constituents nor associated proteins have been identified, leaving its classification unresolved. This limitation is not minor: without molecular identity, key mechanistic claims, such as membrane insertion, curvature induction, and vesicle nucleation, remain untestable hypotheses that currently limit integration into signaling and trafficking regulatory frameworks. The field therefore lacks the minimal molecular grounding required for mechanistic validation.

Technically, resolving this will require integrated strategies combining proximity-labeling proteomics, density-based fractionation, and lipidomic profiling of enriched membrane fractions. Cryo-ET sub-tomogram averaging may provide structural constraints, but cannot resolve molecular identity on its own. Until such data exist, the PND must be considered a structurally defined but molecularly anonymous entity, limiting both mechanistic and translational interpretation in the context of cellular communication biology.

### Temporal blindness and sampling bias

A second major limitation arises from the inherently static nature of cryo-ET. While it provides high-resolution structural snapshots, it cannot resolve temporal progression, directionality, or kinetic transitions. As a result, the proposed sequence, PND insertion, vesicle nucleation, HD expansion, and scission, remains a logically constructed but unverified trajectory. Each observed state may alternatively represent independent, metastable endpoints rather than sequential intermediates.

This limitation is compounded by size constraints: both HD (~ 160 nm) and PND (~ 42 nm) lie below the effective resolution of most live-cell optical systems. Even advanced correlative methods (cryo-CLEM, expansion microscopy) provide spatial correlation but not true dynamic continuity. Therefore, the field currently lacks direct evidence for hemifusome dynamics in living systems and for their potential role in real-time regulation of endosomal communication processes. Resolving this will require next-generation hybrid approaches capable of bridging molecular resolution with real-time imaging.

### Absence of disease-relevant models

Despite strong conceptual links to neurodegeneration and lysosomal storage disorders, hemifusomes have not yet been investigated in disease-relevant biological systems. This creates a critical translational gap: all proposed disease associations remain extrapolations from general endolysosomal dysfunction rather than hemifusome-specific evidence linked to signaling or communication pathway disruption. Without evaluation in patient-derived cells or in vivo models, it is not possible to determine whether hemifusomes are drivers, modifiers, or merely correlated features of pathology.

Closing this gap requires systematic application of cryo-ET and emerging reporter systems in induced pluripotent stem cell (iPSC)-derived neurons and patient fibroblasts, followed by integration with genetic perturbation once molecular components are identified.

## Future directions and research perspectives

### Five foundational priorities

The field of hemifusome biology is currently defined by a limited but clearly identifiable set of unresolved questions. Progress will depend on addressing five interconnected priorities. First, the molecular composition of PNDs must be established, as it underpins all mechanistic models. Second, the temporal dynamics of hemifusome formation and resolution must be directly visualized rather than inferred. Third, causal genetic determinants must be identified to transition from structural description to mechanistic biology. Fourth, the regulation of hemifusome abundance across physiological states must be determined, including whether the reported ~ 10% prevalence is constitutive or adaptive within signaling-active cellular contexts. Finally, the existence and functional relevance of hemifusomes in terminally differentiated cells, particularly neurons, must be directly tested. Together, these priorities define the minimal framework required to transform hemifusome research into a mature biological field with defined relevance to cellular communication systems.

### Decoding PND composition

Resolving PND composition will likely require a staged discovery pipeline rather than a single direct assay. Proximity-labeling strategies using engineered membrane-targeted enzymes may help identify proteins enriched near candidate PND-containing membrane regions; however, in the absence of PND-specific proteins, lipids, or targeting motifs, such approaches would be expected to capture a broader membrane-proximal proteome from the surrounding hemifusion diaphragm and adjacent endosomal compartments. Accordingly, any proximity-labeling signal would require rigorous orthogonal validation to distinguish potential PND-associated components from nonspecific membrane-proximal background. In parallel, density-based lipid enrichment combined with mass spectrometry may provide candidate lipid profiles, but only if the enriched material can first be validated as containing PND-associated structures. Without such validation, co-purifying lipid droplets, membrane fragments, vesicles, or protein aggregates could confound lipidomic interpretation. Thus, these approaches should be viewed as candidate discovery strategies that may generate testable hypotheses rather than definitive methods for establishing PND lipid core composition.

### Capturing live-cell dynamics

A central challenge is bridging structural snapshots with dynamic behavior. Cryo-CLEM provides an immediate bridge between fluorescence localization and ultrastructure, but remains fundamentally static. Emerging imaging modalities, including lattice light-sheet microscopy and expansion-based super-resolution techniques, offer a partial solution by extending temporal resolution while preserving subcellular context. Nevertheless, true mechanistic insight will likely require hybrid approaches that combine live-cell tracking with rapid cryo-fixation. Without such integration, hemifusome dynamics will remain reconstructive rather than directly observed and their role in dynamic endosomal communication will remain unresolved.

### Genetic dissection of biogenesis

Establishing causality in hemifusome formation requires unbiased genetic perturbation strategies. Genome-wide CRISPR-based screens, using hemifusome frequency or validated proxies as readouts, represent the most direct route to identifying essential regulators. In parallel, focused sub-libraries targeting membrane curvature regulators, lipid metabolism enzymes, Rab GTPases, and ESCRT-independent trafficking factors provide a higher-resolution entry point for early mechanistic discovery. The critical outcome of these approaches is not only gene identification but also the establishment of a causal framework linking molecular machinery to hemifusome architecture and its potential regulatory role in endosomal signaling pathways.

### From discovery to clinical translation

Translational progress depends on a strict sequence of validation steps. Molecular identification of PND components is the foundational requirement; without it, no biomarker or therapeutic strategy can be reliably developed. Once defined, PND-associated proteins may serve as indicators of endosomal dysfunction in biofluids, enabling early detection of trafficking-related diseases and potentially reflecting altered cellular communication states. In parallel, patient-derived neuronal models will be essential for testing whether hemifusome dysregulation contributes to disease phenotypes. If causal links are established, hemifusome pathways could become targets for small-molecule modulation, with quantitative imaging serving as a primary readout for therapeutic screening. However, this translational trajectory remains entirely contingent on prior molecular validation.

### A call for integrative science

The complexity of hemifusome biology necessitates a fundamentally interdisciplinary approach. Structural cell biology provides the descriptive framework through cryo-ET, while membrane biophysics explains curvature and energetic constraints. Biochemistry and lipidomics are required for molecular resolution, and synthetic biology offers tools for reconstruction and manipulation.

Equally, nanotechnology provides translational pathways for engineering hemifusome-inspired systems. The discovery of hemifusomes represents a structural breakthrough, but its full significance will only emerge through coordinated integration across disciplines. Without such convergence, the field risks remaining at a purely descriptive stage despite its conceptual potential for understanding and modulating membrane-based cellular communication systems.

## Future perspectives: hypothesis-generating extensions beyond current evidence

The preceding sections synthesize what current structural and biophysical evidence supports regarding hemifusome and PND biology. In this section, we deliberately separate a set of more speculative extensions cargo sorting and cargo transfer models, cytosolic cargo release, and PND-inspired drug delivery from the evidence-based discussion above. These ideas are scientifically motivated by the observed hemifusome architecture, but none has yet been experimentally tested, and they should be read as hypotheses intended to guide future experimental design rather than as conclusions supported by existing data.

### Cargo sorting and cargo transfer: a hypothetical model

The exclusion of transferrin receptor labeling from hemifusome-associated vesicles has been interpreted as evidence of divergence from canonical ESCRT-dependent cargo handling [6, 26]. Extending this observation, it is tempting to hypothesize that hemifusomes constitute a cargo-selective sorting station in which lipidic or non-ubiquitylated material is preferentially partitioned into the nascent vesicle, potentially via a PND-mediated transfer mechanism across the hemifusion diaphragm. However, this remains an entirely untested model: no cargo-tracking, proteomic, or lipidomic study has directly examined material transfer across the HD, and the single negative marker (transferrin receptor) used to infer ESCRT independence cannot, on its own, support a positive claim of selective cargo sorting. We propose this model purely as a testable hypothesis for future proximity-labeling and pulse-chase cargo-tracking studies.

### Cytosolic release as a speculative resolution pathway

The “direct” topological configuration of hemifusomes, in which the smaller vesicle faces the cytoplasm, raises the speculative possibility that HD resolution could, under certain conditions, release vesicular content directly into the cytosol rather than sequestering it intraluminally. If true, this would represent a fundamentally novel route bypassing canonical endosomal-lysosomal degradation. However, no study has observed or measured cytosolic release events, and the energetic and topological requirements for such a transition remain entirely theoretical. We flag this only as a conceptual possibility warranting dedicated live-cell or reporter-based investigation, not as a demonstrated mechanism.

### PND-inspired drug delivery: a long-range translational hypothesis

Building on the structural rationale outlined in Sect.  "Bio-inspired nanoparticle design", "Engineering artificial hemifusomes and PNDs", "Conceptual basis for hemifusome-inspired drug delivery", we speculate that a synthetic system capable of reproducing PND-like membrane insertion and curvature stabilization could, in principle, offer an alternative to conventional lipid-nanoparticle-based endosomal escape strategies. Such a system might theoretically enable tunable, topology-dependent cargo release either intraluminal sequestration or direct cytosolic delivery depending on how the hemifusion intermediate resolves. We emphasize that this remains a purely conceptual extrapolation: no engineered nanoparticle has reproduced PND behavior, no hemifusome-inspired delivery vehicle has been synthesized or tested in any biological system, and the molecular determinants of PND membrane insertion remain unknown. Realizing this possibility will require, at minimum, molecular identification of native PND components before any rational design effort can begin.

### Framing these perspectives

We present Sect.  "Cargo sorting and cargo transfer: a hypothetical model", "Cytosolic release as a speculative resolution pathway", "PND-inspired drug delivery: a long-range translational hypothesis" separately from the evidence-based synthesis in Sects.  "Structural and biophysical characterization of hemifusomes and proteolipid nanodroplets", "Biogenesis of hemifusomes", "Functional implications of hemifusomes in cellular physiology and disease", "Hemifusomes at the interface of nanotechnology and synthetic biology" to avoid conflating structural observation with mechanistic or translational speculation. These perspectives are intended to stimulate hypothesis-driven experimental work particularly cargo-tracking, live-cell dynamics, and synthetic reconstitution studies rather than to imply that cargo sorting, cytosolic release, or PND-based drug delivery are established or even likely functions of hemifusomes at this stage.

### Study limitations

This review is limited by the early stage of hemifusome research, with the available evidence currently originating primarily from a single landmark study by Tavakoli et al. [6] and its associated commentary. Consequently, current mechanistic interpretations remain dependent on a limited experimental framework, and independent replication across laboratories, cell types, and imaging approaches will be essential before broader conclusions regarding hemifusome biology within endosomal communication and signaling networks can be established. In addition, the proposed functional, disease-associated, and translational implications remain largely inferential, arising from structural observations and biological analogy rather than direct experimental validation. Accordingly, these concepts should be considered hypothesis-generating models rather than established roles in membrane trafficking, cellular communication, or therapeutic applications.

Finally, the static nature of cryo-electron tomography limits direct assessment of temporal dynamics, leaving critical aspects such as formation kinetics, regulatory mechanisms, molecular composition, and cell-type specificity unresolved. These limitations currently restrict definitive interpretation of hemifusome behavior and its broader biological significance in dynamic cellular environments.

## Conclusion

The identification of hemifusomes and proteolipid nanodroplets (PNDs) introduces a previously unrecognized level of complexity in membrane remodeling biology. Rather than representing simple transient hemifusion events, hemifusion diaphragms (HDs) appear to form distinct membrane architectures that can persist over the imaging timescale observed in cryo-electron tomography studies. This structural organization is associated with PNDs, which represent morphologically defined but molecularly uncharacterized elements that may contribute to membrane curvature-associated processes and vesicle remodeling. Current evidence is consistent with the possibility that hemifusomes represent a distinct mode of endosomal membrane organization that differs from canonical ESCRT-mediated pathways; however, whether this reflects a truly ESCRT-independent mechanism remains unresolved and requires direct molecular and functional investigation.

Accordingly, hemifusome biology should currently be considered a structurally supported but mechanistically incomplete framework. Proposed roles in MVB maturation, ILV heterogeneity, cargo organization, and exosome-related processes remain hypothesis-generating concepts that require rigorous experimental validation. Likewise, potential disease connections and nanomedicine applications should be regarded as future possibilities rather than established biological or translational functions. Future progress will depend on integrating cryo-electron tomography with molecular composition analysis, genetic perturbation approaches, and dynamic imaging strategies to define the mechanisms governing hemifusome formation, remodeling, and functional significance. In this context, hemifusomes provide a promising system for investigating previously unrecognized principles of membrane organization and vesicle biogenesis.

## Data Availability

No datasets were generated or analysed during the current study.
